# Pharmacologic targeting of renal ischemia-reperfusion injury using a normothermic machine perfusion platform

**DOI:** 10.1038/s41598-020-63687-0

**Published:** 2020-04-24

**Authors:** Ahmer M. Hameed, David B. Lu, Heather Burns, Nicole Byrne, Yi Vee Chew, Sohel Julovi, Kedar Ghimire, Negar Talaei Zanjani, Chow H. P’ng, Daniel Meijles, Suat Dervish, Ross Matthews, Ray Miraziz, Greg O’Grady, Lawrence Yuen, Henry C. Pleass, Natasha M. Rogers, Wayne J. Hawthorne

**Affiliations:** 1 Department of Surgery, Westmead Hospital, Sydney, Australia; 2 Westmead Institute for Medical Research, Sydney, Australia; 3 Sydney Medical School, University of Sydney, Sydney, Australia; 4 Institute for Clinical Pathology and Medical Research, Westmead Hospital, Sydney, Australia; 5 St George’s, University of London, London, UK; 6 Department of Animal Care, Westmead Hospital, Sydney, Australia; 7 Department of Anesthesia, Westmead Hospital, Sydney, Australia; 8 Department of Surgery, The University of Auckland, Auckland, New Zealand; 9 Department of Transplant/Renal Medicine, Westmead Hospital, Sydney, Australia

**Keywords:** Physiology, Translational research, Renal replacement therapy, Urology

## Abstract

Normothermic machine perfusion (NMP) is an emerging modality for kidney preservation prior to transplantation. NMP may allow directed pharmacomodulation of renal ischemia-reperfusion injury (IRI) without the need for systemic donor/recipient therapies. Three proven anti-IRI agents not in widespread clinical use, CD47-blocking antibody (αCD47Ab), soluble complement receptor 1 (sCR1), and recombinant thrombomodulin (rTM), were compared in a murine model of kidney IRI. The most effective agent was then utilized in a custom NMP circuit for the treatment of isolated porcine kidneys, ascertaining the impact of the drug on perfusion and IRI-related parameters. αCD47Ab conferred the greatest protection against IRI in mice after 24 hours. αCD47Ab was therefore chosen as the candidate agent for addition to the NMP circuit. CD47 receptor binding was demonstrated by immunofluorescence. Renal perfusion/flow improved with CD47 blockade, with a corresponding reduction in oxidative stress and histologic damage compared to untreated NMP kidneys. Tubular and glomerular functional parameters were not significantly impacted by αCD47Ab treatment during NMP. In a murine renal IRI model, αCD47Ab was confirmed as a superior anti-IRI agent compared to therapies targeting other pathways. NMP enabled effective, direct delivery of this drug to porcine kidneys, although further efficacy needs to be proven in the transplantation setting.

## Introduction

End-stage renal disease (ESRD) has a sizeable global burden of disease, causing at least 1.2 million global annual deaths^1^. Kidney transplantation is the best available treatment for ESRD, conferring a significant survival benefit over dialysis^2–4^. However, there is a perpetual supply-demand gap between patients awaiting transplantation and the availability of deceased donor kidneys. This has necessitated expansion of the donor pool to include more marginal organs, including donation after circulatory death (DCD) kidneys, which are subjected to greater warm ischemia^5–7^. Short-term transplantation outcomes, including delayed graft function (DGF), are inferior in DCD kidneys in comparison to kidneys from brain-dead (DBD) donors with no significant comorbidities^6^. This increased susceptibility to ischemia-reperfusion injury (IRI) and DGF can translate into poorer long-term graft survival^8^.

As such, an improved method of kidney assessment, repair and preservation is required above and beyond the currently accepted gold standard of cold static storage (CS), particularly in this donor kidney subset. Machine perfusion (MP) preservation is an important alternative that has regained prominence^9^. Normothermic MP (NMP) is especially promising, and is now the subject of a multi-center randomized control trial (RCT) comparing it to CS alone in DCD kidneys^10–13^.

Most pharmacotherapeutics shown to ameliorate renal IRI have been unable to bridge the ‘valley of death’ (translational gap) to the clinic. This is at least partly attributable to the inherent difficulties and ethical considerations associated with the systemic use of such therapies in donors or recipients^14,15^. NMP can serve as a bridge across this valley by providing a platform for direct, non-systemic drug treatment of the kidney whilst it is undergoing normal metabolic processes^15,16^.

Amongst the multiple anti-IRI agents tested in pre-clinical models, CD47-blocking antibody (αCD47Ab), recombinant thrombomodulin (rTM), and soluble complement receptor 1 (sCR1) are especially translatable as they have been safely employed for other clinical applications^17–25^. However, the comparative efficacy of these agents has not been established. αCD47Ab ameliorates thrombospondin (TSP)-1 mediated IRI signaling, including inhibition of nitric oxide and promotion of oxidative stress^21^. sCR1 is an inhibitor of the classical and alternative complement pathways, activation of which is important in IRI^26^. rTM is an anti-coagulant molecule involved in the generation of activated protein C, although its efficacy in IRI may be more attributable to anti-inflammatory effects^17,27^. Because IRI is characterized by the activation of multiple intersecting pathways^28,29^, it is also plausible that synergistic anti-IRI effects may be derived by delivering two or more of these agents together.

The aims of this study were to directly compare the acute effects of αCD47Ab, sCR1, and rTM in a murine model of renal IRI and to establish the combined efficacy of two of the best agents. We then show that the chosen drug could be directly delivered to porcine DCD kidneys using NMP to enhance renal perfusion parameters and ameliorate IRI. The primary focus of this study is the immediate phase of IRI, which correlates to the immediate post-transplant setting and the risk of delayed graft function in higher risk renal allografts.

## Methods

### Animal work – ethics

All protocols were approved by the Western Sydney Local Health District Animal Ethics Committee, in accordance with the Australian code for the care and use of animals for scientific purposes (8^th^ Ed., 2013), developed by the National Health and Medical Research Council. Animal experiments adhere to the ARRIVE guidelines.

### PART 1. Comparison of IRI targets – murine model

#### Animals

Male C57BL/6 mice (10–12 weeks age) were obtained from the Animal Resources Centre (Canning Vale, Australia), acclimatized and allowed free access to food and water until surgery. The use male C57BL/6 mice is commonly accepted and performed in kidney IRI experiments^30,31^.

#### IRI model

General anesthesia (GA) was induced using intra-peritoneal ketamine (100 mg/kg) and xylazine (8 mg/kg). The abdomen was shaved, prepared with povidone-iodine, and a midline laparotomy performed. The small intestine was wrapped in gauze moistened with 3 ml of 0.9% sodium chloride (NaCl) (36–37 °C), and placed outside the operating field. The right renal pedicle was ligated (6–0 silk tie) prior to a right nephrectomy. Each drug/combination was diluted to a total volume of 0.25 ml in 0.9% NaCl, and injected intra-venously (inferior vena cava) using a 30 G needle. The left renal pedicle was then clamped for 25 minutes using an arterial microvascular clamp (Roboz Surgical Instrument Co., MD, USA). Mouse temperature was maintained at 36 °C (RightTemp Temperature Monitor and Homeothermic Warming Control Module [Kent Scientific, CT, USA]). Kidney reperfusion was confirmed by the return of its original color. Warmed 0.9% NaCl (0.3 ml) was instilled into the peritoneal cavity, abdominal contents were replaced anatomically, and the defect was closed using 6–0 PDS. Sham-operated mice (n = 3) underwent laparotomy and subsequent closure after an interval of 25 minutes, without the induction of IRI. Ethical considerations precluded the use of a larger number of sham mice.

All mice were given buprenorphine (0.1 mg/kg) subcutaneously at defined intervals post-operatively (at least 2 doses). Mice were euthanized 24 hrs after induction of IRI for the collection of all blood and renal tissue samples as this is the time of peak IRI^32–34^.

#### Study groups and pharmacotherapeutic agents

Excluding the sham group, mice were randomized and treated with the following agent(s):

1. Group I – 0.9% NaCl (vehicle control) only
2. Group II – rTM (Asahi Kasei Pharma Co., Tokyo, Japan), 1 mg/kg body weight^35^
3. Group III –sCR1 (CDX-1135; Celldex Therapeutics, MA, USA), 25 μg/g body weight^36^
4. Group IV – αCD47Ab (MIAP 301 [sc-12731]; Santa Cruz Biotechnology, TX, USA), 0.8 μg/g body weight^30^
5. Group V – combination of best 2 performing drugs, determined by relative serum creatinine decrease compared to vehicle controls – αCD47Ab (0.8 μg/g body weight) and sCR1 (25 μg/g body weight) given as a single combined dose.

#### Blood and renal tissue samples

Mice were culled 24 hours after reperfusion under GA. Blood samples were taken from the IVC immediately prior to exsanguination. The left kidney was removed and processed – samples were stored in 10% formalin, RNA*later* RNA stabilization solution (Ambion/Thermo Fisher Scientific, TX, USA), and also snap frozen in dry ice (with or without OCT media [Tissue-Tek, ProSciTech, Australia]). Serum samples were analyzed for blood urea nitrogen (BUN) and creatinine levels using the Dimension Vista 1500 Lab System (Siemens, Munich, Germany).

#### Histology – hematoxylin and eosin (H&E)

Paraffin-embedded sections (6 μm thickness) were stained with H&E. Renal damage at the corticomedullary junction was scored by 2 blinded renal histopathologists. Six regions of interest were taken per section, and tubular damage was scored from 0–5 (0 – no tubules affected; 1 – 1-10% of tubules; 2 – 11-25% of tubules; 3–26–45% of tubules; 4–46–75% of tubules; 5 –>75% of tubules) as described previously^21^.

#### Immunohistochemistry

Immunohistochemistry was performed using the Leica Bond Rx Automated Research Stainer (Leica Biosystems, Wetzlar, Germany) and the Bond Polymer Refine Detection Kit (Leica Biosystems, Newcastle upon Tyne, UK), on formalin-fixed, paraffin-embedded sections (6 μm). An optimized staining protocol was developed – 3–4% hydrogen peroxide block (20 mins), primary antibody (60 mins) and secondary antibody (30 mins) incubation, administration of poly-HRP IgG reagent for localization of rabbit (secondary) antibodies (8 mins), application of 3,3’-Diaminobenzidine tetrahydrochloride hydrate (DAB) (5 mins), and hematoxylin counterstaining (5 mins). Slides were cover-slipped using mounting media (Dako/Agilent Technologies, CA, USA). Neutrophils were detected using primary rat anti-mouse Ly-6G/Ly-6C antibody (RB6-8C5) at a 1:200 dilution (Biolegend, CA, USA), and secondary rabbit anti-rat IgG (BA-4001) at a 1:200 dilution (Vector Laboratories, CA, USA). Positively stained cells were counted from 5 high-power fields (HPF) at the corticomedullary junction in each section. Kim-1 was detected using anti-rabbit Kim-1 antibody (E1R9N) at 1:100 dilution (Cell Signaling Technology (Danvers, MA), and secondary goat anti-rabbit IgG (BA-1000) at a 1:200 dilution (Vector Laboratories). Intensity of staining was calculated using ImageJ.

#### Reactive oxygen species (ROS) characterization – cytochrome C and Amplex Red

##### Measurement of Superoxide (O2•^−^) in particulate fractions using cytochrome c:

Whole kidney tissue was homogenized in ice-cold phosphate buffer (PBS) and scraped in lysis buffer (8 mM potassium, sodium phosphate buffer pH 7.0, 131 mM NaCl, 340 mM sucrose, 2 mM NaN3, 5 mM MgCl2, 1 mM EGTA, 1 mM EDTA and protease inhibitors [Roche Diagnostics GmbH, Mannheim, Germany]). Tissue was further lysed by five freeze/thaw cycles, and passage through a 30-gauge (G) needle 5 times. The lysate was centrifuged at 1000 g (5 min; 4 °C) to remove unbroken cells, nuclei and debris. Extreme care was taken to maintain the lysate at a temperature close to 0 °C. The cell lysate was centrifuged at 28,000 g (15 min; 4 °C). The supernatant was removed, membranes were resuspended in lysis buffer, and protein concentration was measured using the Bradford microplate method.

Superoxide production in particulate fractions (20 μg/ml) of untreated, αCD47Ab-, rTM-, or sCR1-treated mice was measured in 0.1 ml of oxidase assay buffer (65 mM sodium phosphate buffer pH 7.0, 1 mM EGTA, 10 μM FAD, 1 mM MgCl2, 2 mM NaN3 and 0.2 mM cytochrome c [Sigma-Aldrich]). Superoxide production was initiated by the addition of 180 μM NADPH and was calculated from the initial linear rate of superoxide dismutase (SOD) (150 U/ml) (Sigma-Aldrich) inhibitable cytochrome c reduction quantified at 550 nm using an extinction coefficient of 21.1 mM-1 cm-1 (Biotek Synergy 4 Hybrid Multi-Mode Microplate Reader).

##### Hydrogen peroxide (H_2_O_2_)-generating activity:

Whole kidney tissue was homogenized in ice-cold disruption buffer (PBS containing 0.1 mM EDTA, 10% glycerol, protease inhibitor cocktail, and 0.1 mM phenylmethylsulfonyl fluoride [Sigma-Aldrich]), and further lysed as for superoxide. Lysate (50 μg/ml) was added to the assay mixture (25 mM Hepes, pH 7.4, containing 0.12 M NaCl, 3 mM KCl, 1 mM MgCl2, 0.1 mM Amplex red [Invitrogen, CA, USA], and 0.32 U/ml HRP). The reaction was initiated by the addition of 36 μM NADPH. Fluorescence measurements were made using a Biotek Synergy 4 hybrid multimode microplate reader with a 530/25-excitation and a 590/35-emission filter. The reaction was monitored at 25 °C (15 min); the emission increase was linear during this interval. To confirm the H_2_O_2_ signal, catalase (300 U/ml; Sigma-Aldrich) was added in parallel wells, and the catalase-inhibitable rate of H_2_O_2_ production was quantified from an H_2_O_2_ standard curve.

#### Inflammatory and injury markers – pro-inflammatory cytokine/chemokine mRNA expression

Kidney tissue sections stored in RNA*later* were homogenized, and RNA was extracted using the ISOLATE II RNA Mini Kit as per manufacturer’s instructions (Bioline/Meridian Life Science, TN, USA). One microgram of RNA was reverse transcribed using the SensiFAST cDNA synthesis kit (Bioline/Meridian Life Science). cDNA amplification was performed in triplicate in volumes of 10 μL, consisting of cDNA, SensiFAST Probe No-ROX, and the relevant gene-specific primer/Taqman™ probes (HPRT1 – MM00446968_m1; IL-6 – MM00446190_m1; TNF-α – MM00443258_m1; IL-1β – MM00434228_m1; CCL2 – MM00441242_m1; CXCL2 – MM00436450_m1; KIM-1 – MM00506686_m1) (Thermo Fisher Scientific, MA, USA). Real-time polymerase chain reaction (RT-PCR) was performed on a Bio-Rad CFX384 machine – 95 °C for 10 mins, 95 °C for 30 sec (40 cycles), and 60 °C for 45 sec (40 cycles). The ∆∆Ct method was used to calculate expression fold changes normalized to HPRT1, with the 0.9% NaCl group utilized as the control.

#### Immunofluorescence

Complement C3 and C9 staining was ascertained using immunofluorescence. Cryosections (7 μm thickness) were fixed for 10 mins using 4% paraformaldehyde, followed by blocking at room temperature with 1% BSA, 0.1% Tween 20, and 22.5 mg/ml glycine in PBS (30 minutes). The primary antibody of interest (complement C3 [Thermo Fisher Scientific] or C9 polyclonal antibodies [Abcam, Cambridge, UK]) was added to separate cryosections at a 1:250 dilution in blocking solution, and left in a humidified chamber overnight (4 °C). Sections were incubated with goat anti-rabbit Alexa Fluor 647 secondary antibody (Thermo Fisher Scientific) at a 1:400 dilution at room temperature (1 hour), co-stained with DAPI (1 min), and then cover-slipped. Staining was visualized using a confocal microscope, and quantified using Image J.

#### TUNEL staining

Cellular death was ascertained using terminal deoxynucleotidyl transferase-mediated dUTP nick-end labeling (TUNEL) staining, performed with a commercially available kit (*In Situ* Cell Death Detection Kit, TMR Red; Sigma-Aldrich/Merck, MO, USA), as per the manufacturer’s instructions. Staining was visualized by confocal microscopy; TUNEL-positive cells were counted from 3–5 HPF in each section.

### PART 2. Direct intra-renal delivery of αCD47Ab using NMP – porcine DCD model

In this part, porcine kidneys undergoing ‘standard’ NMP were compared to kidneys undergoing NMP with the addition of αCD47Ab (the best performing anti-IRI agent from the murine study).

#### Animals

Female adult outbred Landrace pigs (70.7 ± 14.2 kg) obtained from a certified animal supplier were acclimatized and allowed free access to food until 12 hours before surgery. Water was available *ad libitum* until the time of surgery. Outbred pig kidneys provide an ideal pre-clinical model in kidney transplantation-related studies^37–39^.

#### Porcine kidney retrieval – DCD model

Kidney retrieval and a DCD model was established as previously described^40^. All operative procedures were performed under general anesthetic, including pre-medication with intramuscular 1 mg/kg Ilium Xylazil (Xylazine, Troy Laboratories Pty Ltd, Sydney, Australia), 25 mg/kg Tiletamine combined with 25 mg/kg Zolazepam (Zoletil 100, Virbac Australia Pty Ltd, Sydney, Australia), and 1 mg/kg Azaperone (Stresnil, Boehringer Ingelheim Pty Ltd, Sydney, Australia). General anesthesia was subsequently induced with 16 mg/kg intravenous thiopentone (Pentothal, Abbott Australasia Pty Ltd., Sydney, Australia) and maintained with 1–2% isoflurane (Zeneca Ltd., Macclesfield, UK) in oxygen after intubation. NaCl (0.9%) was given intravenously at 60 ml/hr for the surgical duration. After a midline laparotomy, the renal pedicles and aorta were exposed/mobilized. The infra-renal aorta was cannulated using a TUR giving set (Baxter Healthcare, IL, USA), through which each pig was exsanguinated; blood was collected into tubes containing Anticoagulant-Citrate-Dextrose solution A (ACD-A) (Aurora Bioscience, Bella Vista, Australia)^41^. During exsanguination, the renal pedicle was clamped for 10 mins to simulate warm ischemia in a DCD setting. The renal artery was cannulated intra-corporeally using heparin tips cannulas (Medtronic, Minneapolis, USA), and the ureter was cannulated using a 12 G intra-venous catheter (Terumo Surflo Catheters, Tokyo, Japan). After exactly 10 mins, the kidney was cold-perfused via the renal artery with 500 ml University of Wisconsin (UW) solution (Bridge to Life, South Carolina, USA) containing 10,000 IU/L heparin. The two experimental groups were – (i) control kidneys (no further additives); (ii) treatment kidneys (given the best performing anti-IRI agent from the murine study, i.e. [porcine/human-specific] αCD47Ab – BRIC-126 [sc-59079], Santa Cruz Biotechnology) via the renal artery (100 μg diluted in 10 ml UW solution), immediately after the initial UW flush. All kidneys were stored in UW solution prior to NMP (at 4 °C for 6 hrs).

#### Normothermic machine perfusion

Kidney NMP was performed using a modified cardio-pulmonary bypass circuit, as described previously^40^. In brief, packed red blood cells (PRBCs) were isolated from autologous whole blood, and leucocyte-depleted using a leucocyte filter (Imugard III-RC, Terumo, Tokyo, Japan). PRBCs (230 ml) were added to a reservoir (with integrated oxygenator, heat exchanger, and arterial filter) (Terumo Capiox FX05, Tokyo, Japan), along with 150 ml Hartmann’s solution, 250 ml Gelofusine (B. Braun Australia Pty Ltd, Bella Vista, Australia), 18 ml sodium bicarbonate 8.4%, 50 ml mannitol 10%, 2000 IU of unfractionated heparin, 5 ml calcium gluconate 0.22 mmol/ml, and 25 ml water for injection. Creatinine (Merck, Darmstadt, Germany) was added to achieve a concentration of 1000 μmol/L to allow for subsequent creatinine clearance (CrCl) calculation^42^.

The kidney was flushed with Hartmann’s solution to remove residual UW solution, weighed, and perfused through the renal artery at a mean pressure of 75–85 mmHg and temperature of 37 °C (1 hr). The 1 hour time period was chosen as it has been shown to be effective in human kidney transplantation after initial CS, and is now the subject of a multi-center RCT in the UK^11,12^. The kidney was placed in a customized 3D-printed copolyester perfusion chamber during NMP^43^. Continuous infusions of verapamil (0.5 mg/hr), 5% dextrose (5 ml/hr), and M199 nutrient solution containing 100 IU of actrapid and multivitamins (1 vial of Soluvit N dissolved in Vitalipid N; Fresenius Kabi, Bad Homburg, Germany) (100 ml at 20 ml/hr) were also provided. Immediately prior to starting NMP in treatment kidneys, 200 μg of αCD47Ab (BRIC-126) was directly injected into the renal arterial line (i.e. ~0.8 μg/g of kidney weight).

Overall, the two comparator NMP groups were as follows:

1. Group I – αCD47Ab-treatment group: cold UW flush containing αCD47Ab → 6 hr CS → 1 hr NMP with further addition of αCD47Ab
2. Group II – No αCD47Ab-treatment (control group): cold UW flush → 6 hr CS → 1 hr NMP (standard, without addition of αCD47Ab).

#### Renal tissue, blood, and urine samples

Wedge kidney biopsies were obtained from the renal cortex for both study groups at the end of NMP, i.e. after 1 hr of NMP. Perfusate blood samples taken from the arterial arm of the circuit (immediately after commencement, and just prior to cessation, of NMP) were analyzed for sodium (Na), creatinine, lactate dehydrogenase (LDH), and aspartate aminotransferase (AST). Urine samples were taken at the end of NMP and analyzed for creatinine and Na. All automated analyses were conducted using the Dimension Vista 1500 Lab System (Siemens). Blood gas analyses (arterial and venous) for pH, partial pressure of oxygen and carbon dioxide, base excess (BE), lactate, and bicarbonate levels were also conducted at the start and end of NMP using the i-STAT Alinity (Abbott, IL, USA).

#### Calculations

Renal blood flow (RBF) was adjusted to a kidney weight of 250 g and recorded at 5 min intervals. Intra-renal resistance (IRR; pressure/flow) was also calculated at each corresponding time point. Urine output (UO) was measured at the end of NMP. CrCl, fractional excretion of sodium (FeNa), and renal oxygen consumption were calculated as described elsewhere^42^.

#### Histology

H&E was performed as above. Sections were scored from 0–3 (from least to most severe) by a blinded renal histopathologist based on the extent of tubular dilatation, tubular debris, cytoplasmic vacuolation, and inflammatory cell infiltration^37,44^.

#### Inflammatory markers – pro-inflammatory cytokine/chemokine mRNA expression

RT-PCR was performed as described above using the following porcine-specific primers: HPRT1 (Ss03388274_m1), IL-6 (Ss03384604_u1), TNF-α (Ss03391318_g1), IL-1β (Ss03393804_m1), and IL-18 (Ss03391203_m1) (Thermo Fisher Scientific).

### CD47 antibody binding to renal tissue – immunofluorescence

αCD47Ab binding to porcine renal tissue was visualized on cryosections fixed with 96% ethanol (room temperature), permeabilized using 0.1% Triton X-100 in PBS (10 minutes), and blocked using 1% BSA and 22.5 mg/ml glycine in PBS (25 minutes). CD47 BRIC-126 is a mouse monoclonal antibody; goat anti-mouse secondary antibody conjugated to Alexa Fluor 647 dye (Thermo Fisher Scientific) was therefore added to the sections (1:400 dilution), and left in a humidified chamber (45 mins). Samples were co-stained with DAPI, and cover-slipped. Fluorescence signaling was visualized using confocal microscopy.

### Renal oxidative stress

Porcine renal tissue oxidative stress was quantified using dihydroethidium (DHE) (Thermo Fisher Scientific), indicative of tissue levels of superoxide. DHE (10 μM in PBS) was added to unfixed cryosections at 37 °C in a light-protected humidified chamber (22 min). Slides were co-stained with DAPI and mounted. Fluorescence was visualized using confocal microscopy. DHE staining density was quantified using Image J software.

#### TUNEL staining

TUNEL staining was performed as described above.

#### Data and statistical analysis

Data is presented as mean ± standard deviation (SD). Continuous parametric variables were compared using the unpaired student’s t-test. In the event that more than two groups of parametric variables were to be compared, the ANOVA test was utilized. Area under the curve (AUC) was calculated for RBF and IRR prior to further statistical comparisons. GraphPad Prism v. 7.02 was used for all statistical analyses. A p-value of <0.05 was deemed statistically significant. The data and statistical analysis comply with the recommendations on experimental design and analysis in pharmacology^45^.

#### Materials

Drugs utilized in this study were obtained as follows – rTM (Asahi Kasei Pharma Co., Tokyo, Japan); and sCR1 (CDX-1135; Celldex Therapeutics, MA, USA); αCD47Ab (murine-specific: MIAP 301 [sc-12731]; porcine-specific: BRIC-126 [sc-59079]; Santa Cruz Biotechnology, TX, USA).

Other major reagents were obtained from the following sources – RNA*later* solution (Ambion/Thermo Fisher Scientific, TX, USA); OCT media (Tissue-Tek, ProSciTech, Australia); Bond Polymer Refine Detection Kit (Leica Biosystems, Newcastle upon Tyne, UK); immunofluorescence mounting media (Dako/Agilent Technologies, CA, USA); rat anti-mouse Ly-6G/Ly-6C antibody (RB6-8C5) (Biolegend, CA, USA); rabbit anti-rat IgG (BA-4001) (Vector Laboratories, CA, USA); RNA extraction kit (Bioline/Meridian Life Science, TN, USA); gene-specific primers (Thermo Fisher Scientific, MA, USA); complement C3 antibody (Thermo Fisher Scientific);vC9 polyclonal antibodies (Abcam, Cambridge, UK); goat anti-rabbit Alexa Fluor 647 secondary antibody (Thermo Fisher Scientific); TUNEL staining kit (*In Situ* Cell Death Detection Kit, TMR Red; Sigma-Aldrich/Merck, MO, USA); University of Wisconsin (UW) solution (Bridge to Life, SC, USA); creatinine (Merck, Darmstadt, Germany); and DHE (Thermo Fisher Scientific)

## Results

### Part 1 – murine renal IRI model (Warm Ischemia)

#### αCD47Ab results in the greatest protection from injury in a murine model of severe IRI

**Figure 1:**
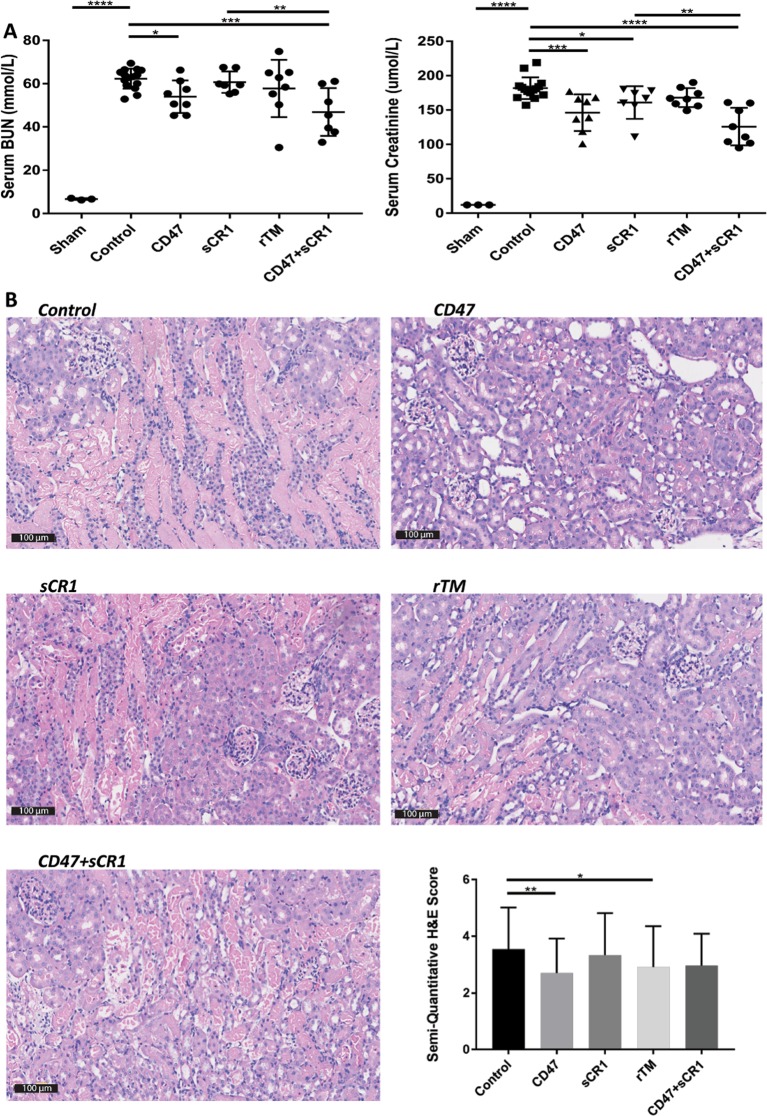
(**A**) Serum BUN and creatinine 24 hours post-IRI in sham mice (n = 3), and mice treated with 0.9% NaCl alone (vehicle control [“control”]) (n = 12), αCD47Ab, sCR1, rTM, or αCD47Ab + sCR1 (n = 8/group). (**B**) Representative H&E sections and semi-quantitative renal tubular damage scores from each treatment group 24 hrs after the induction of IRI (20×). Data shown as mean ± SD. *p < 0.05, **p < 0.01, ***p < 0.001, ****p < 0.0001. n.b. There is more data from controls as at least 1–2 vehicle controls were included during each IRI surgical day to ensure consistency.

Severe IRI was evident in vehicle control murine kidneys 24 hours after induction of ischemia, as indicated by serum BUN and creatinine levels, and the degree of histologic injury seen at the corticomedullary junction (Fig. 1). As expected, sham-operated mice had no significant renal injury, in concordance with other studies^21^. Treatment with αCD47Ab prior to IRI resulted in a significantly lower serum BUN and creatinine, and less histologic damage. A significant decrease in serum creatinine was also seen in the sCR1 alone group, but not the rTM-treated mice. In contrast, rTM-treated mice had significantly less injury evident on histology as compared to controls, but this was not evident in the sCR1 group.

#### Combination of αCD47Ab and sCR1 does not significantly ameliorate IRI in comparison to CD47 alone

Although mice treated with both αCD47Ab and sCR1 (αCD47Ab + sCR1) showed a significant reduction in serum BUN and creatinine in comparison to controls, this decline was not cumulative to that seen with αCD47Ab alone (Fig. 1A). Microscopic (tubular) injury in the αCD47Ab + sCR1 mice was not significantly reduced (Fig. 1B).

#### Neutrophil influx after IRI is depleted in all treatment groups, in particular αCD47Ab and sCR1 given alone

**Figure 2:**
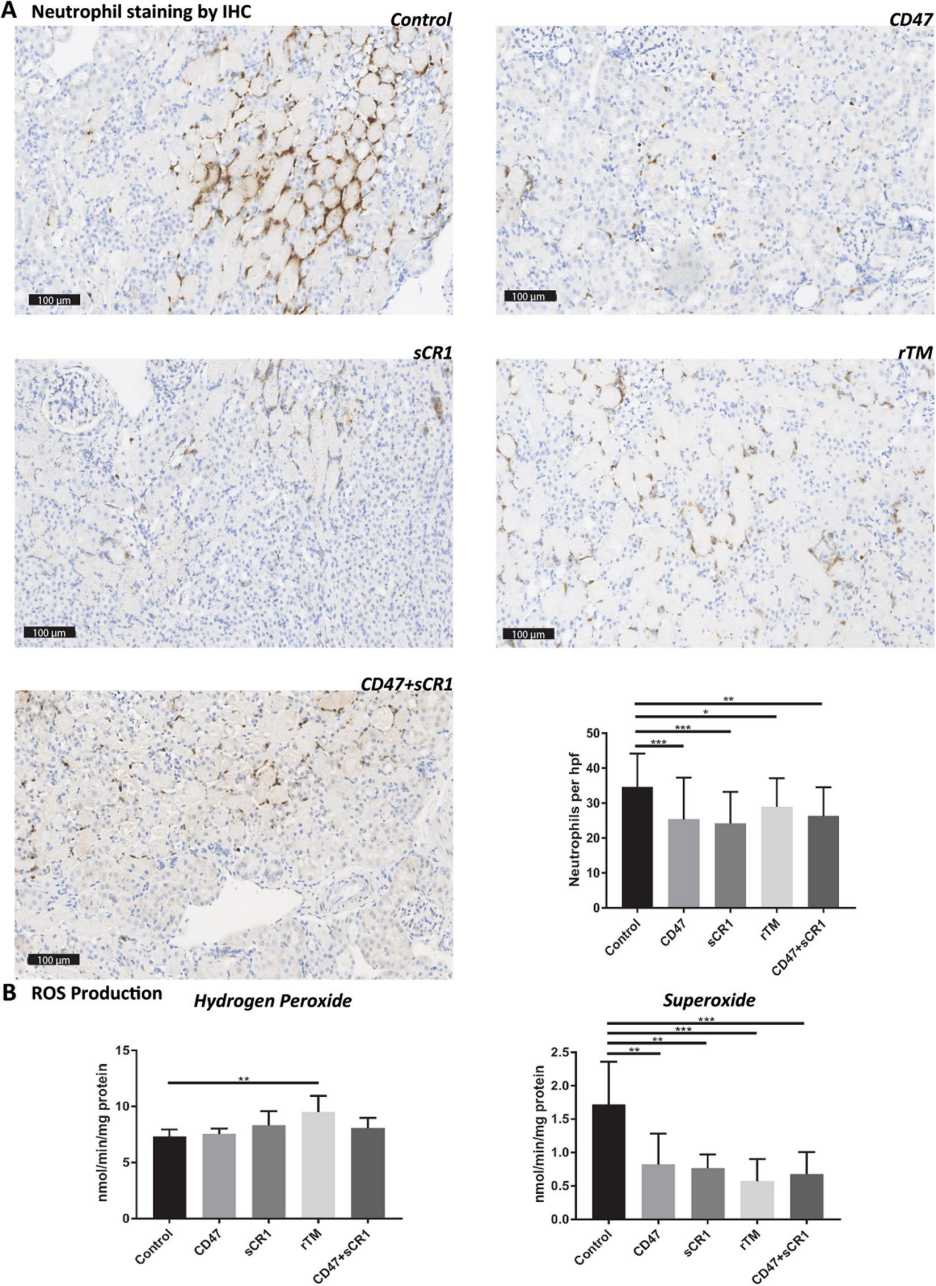
(**A**) Representative sections and quantitative analyses from each murine treatment group after immunohistochemical staining for neutrophils (number of cells per high power field [HPF]) (20×). (**B**) Quantification of reactive oxygen species production (hydrogen peroxide [amplex red] and superoxide [cytochrome **C**]) in all murine treatment groups. Data shown as mean ± SD; n = 5/group. *p < 0.05, **p < 0.01, ***p < 0.001.

Leukocytes, especially neutrophils, infiltrate renal tissue after IRI. Extensive neutrophil infiltration was seen in vehicle controls (Fig. 2A). In comparison, all mouse treatment groups showed significantly less neutrophil staining, with the greatest reduction evident in the sCR1 and αCD47Ab groups of mice.

#### Superoxide but not hydrogen peroxide ROS production is diminished in all treatment groups

Superoxide production was significantly reduced in mice treated with αCD47Ab, sCR1, rTM, or αCD47Ab + sCR1 (Fig. 2B). However, hydrogen peroxide levels did not decrease in any treatment group, and were significantly higher in rTM-treated mice (Fig. 2B).

#### Pro-inflammatory cytokine and chemokine mRNA expression is variably modulated in treated mice after IRI

**Figure 3:**
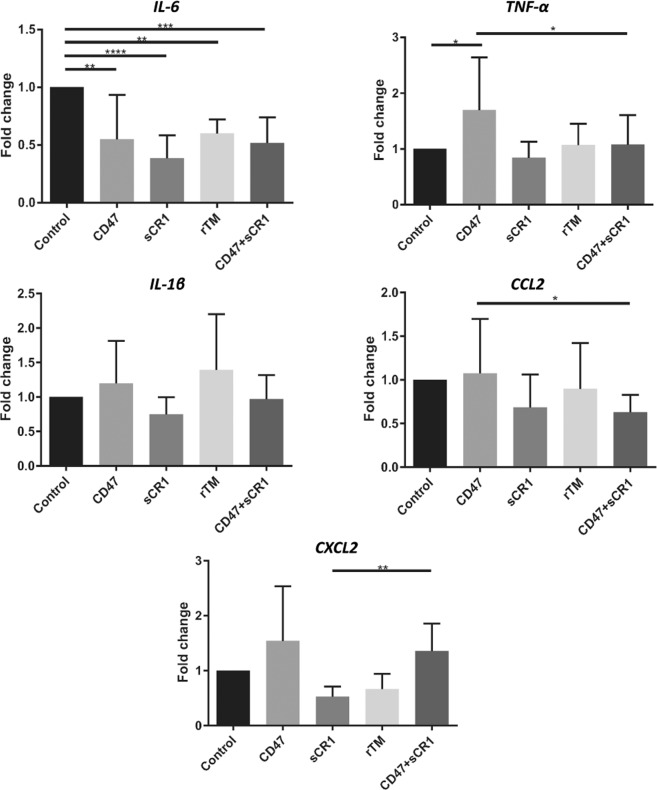
Pro-inflammatory cytokine and chemokine (IL-6, TNF-α, IL-1β, CCL2, and CXCL2) mRNA expression profiles in mouse kidney tissue 24 hrs after the induction of IRI following various drug treatments. Fold change calculated by normalizing to HPRT1, with the 0.9% NaCl (control) mice used as the reference group. Data shown as mean ± SD; n = 5/group. (**B**) *p < 0.05, **p < 0.01, ***p < 0.001, ****p < 0.0001.

**Figure 4:**
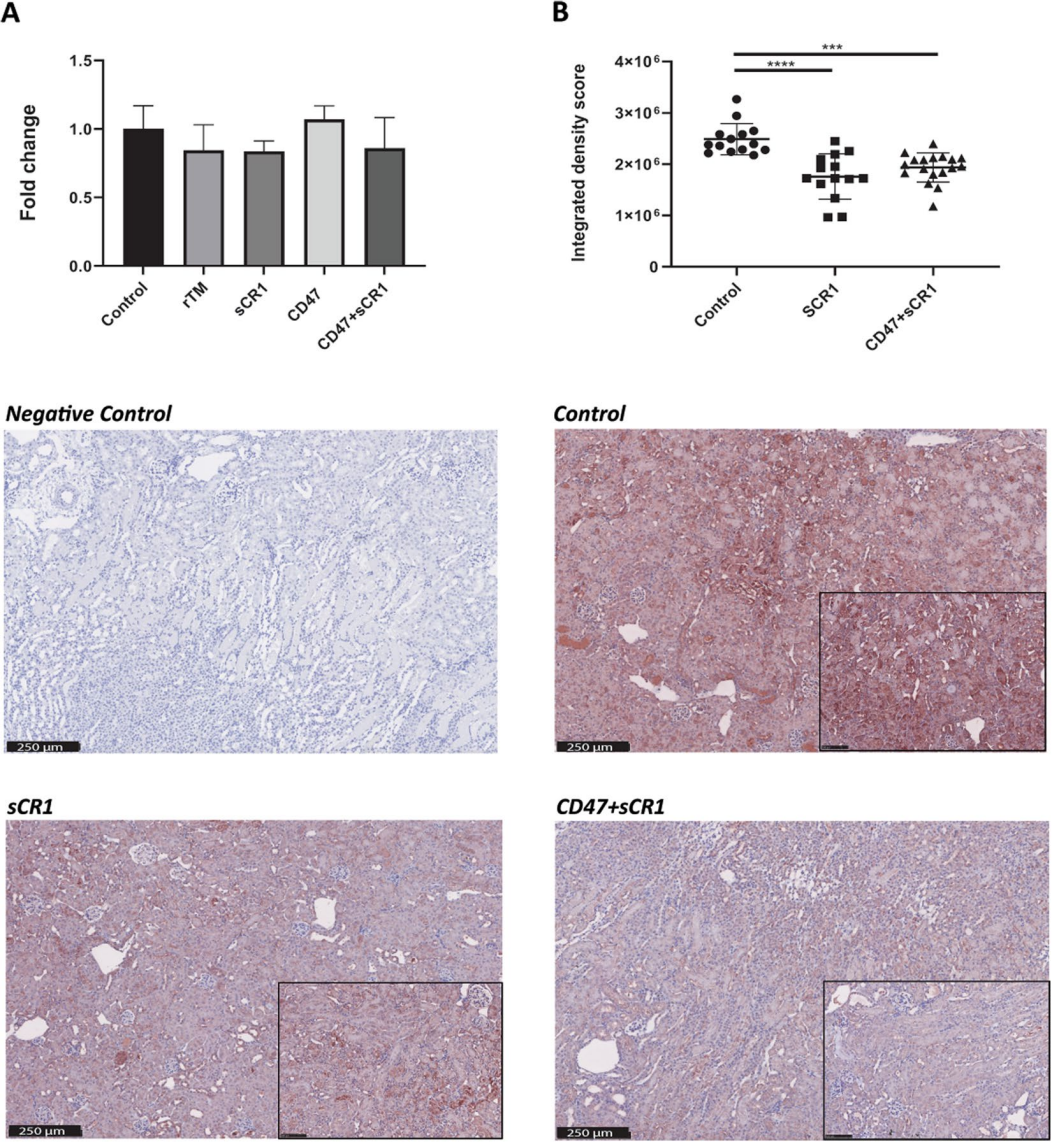
KIM-1 (**A**) mRNA expression and (**B**) immunohistochemical staining in mice 24 hrs after renal IRI. Data shown as mean ± SD; n = 6/group, with insets displayed at 20x zoom with 100 µm scale bars. ***p < 0.001, ****p < 0.0001.

IL-6 levels were significantly lower in all treatment mouse groups at 24 hours in comparison to controls (Fig. 3). However, no significant reductions were seen in the mRNA expression profiles of TNF-α, IL-1β, CCL2, or CXCL2. Furthermore, there was no significant reductions in KIM-1 mRNA expression in any treatment group compared to controls (Fig. 4A).

#### KIM-1 expression is modulated by ameliorating IRI

KIM-1 immunostaining was tested in mice treated with sCR1 or combined sCR1 and αCD47Ab (Fig. 4B); other groups were not tested due to no obvious decrease in mRNA expression profiles by qPCR. Both treatment groups showed significantly reduced KIM-1 staining compared to control mice.

#### Complement C3, but not C9 deposition is reduced by αCD47Ab and/or sCR1 treatment

**Figure 5:**
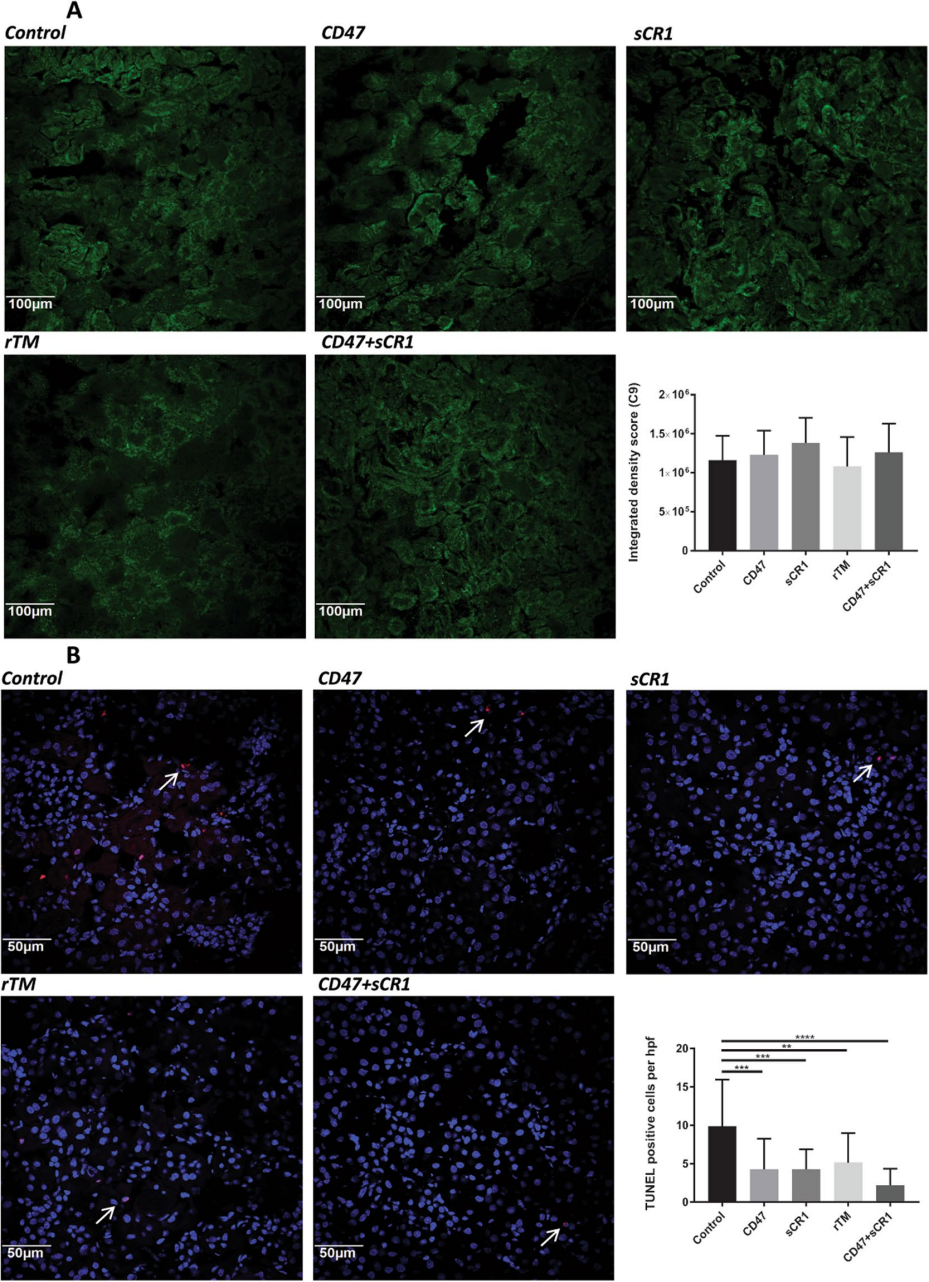
(**A**) Representative renal tissue photomicrographs (immunofluorescence) and quantitative integrated density scores for complement C9 staining 24 hrs post-IRI in each mouse treatment group (20×). (**B**) Quantification of renal cellular death by TUNEL staining, with associated representative photomicrographs (immunofluorescence) (40×). Data shown as mean ± SD; n = 5/group. **p < 0.01, ***p < 0.001, ****p < 0.0001.

All three complement pathways are implicated in IRI, with the activation of C3 and culminating in the formation of the membrane attack complex (C5b-9). No significant differences were seen between any mouse groups with respect to C9 staining (Fig. 5A). C3 deposition was poorly defined, with significant autofluorescence visible; however, it was significantly reduced in all treatment groups except rTM (see figure, supplemental digital content 1).

#### Cell death is reduced in all mice treatment groups in comparison to controls

Renal tubular epithelial cells are the primary site of injury following IRI. Cell death quantified by TUNEL staining 24 hours post-IRI induction was most significantly reduced in αCD47Ab + sCR1-treated mice, although the reduction in the combined blockade group was not significantly greater than that achieved by αCD47Ab or sCR1 treatments alone (Fig. 5B).

### Part 2 – porcine renal DCD model and drug delivery via NMP

As shown in Part 1, αCD47Ab was the most effective treatment in the murine IRI model, and was therefore chosen as the targeted agent for the next study. NMP of porcine kidneys was compared with/without αCD47Ab treatment after a 10 min warm and 6 hr cold ischemic interval.

#### αCD47Ab can be directly and effectively delivered to the kidney using NMP

**Figure 6:**
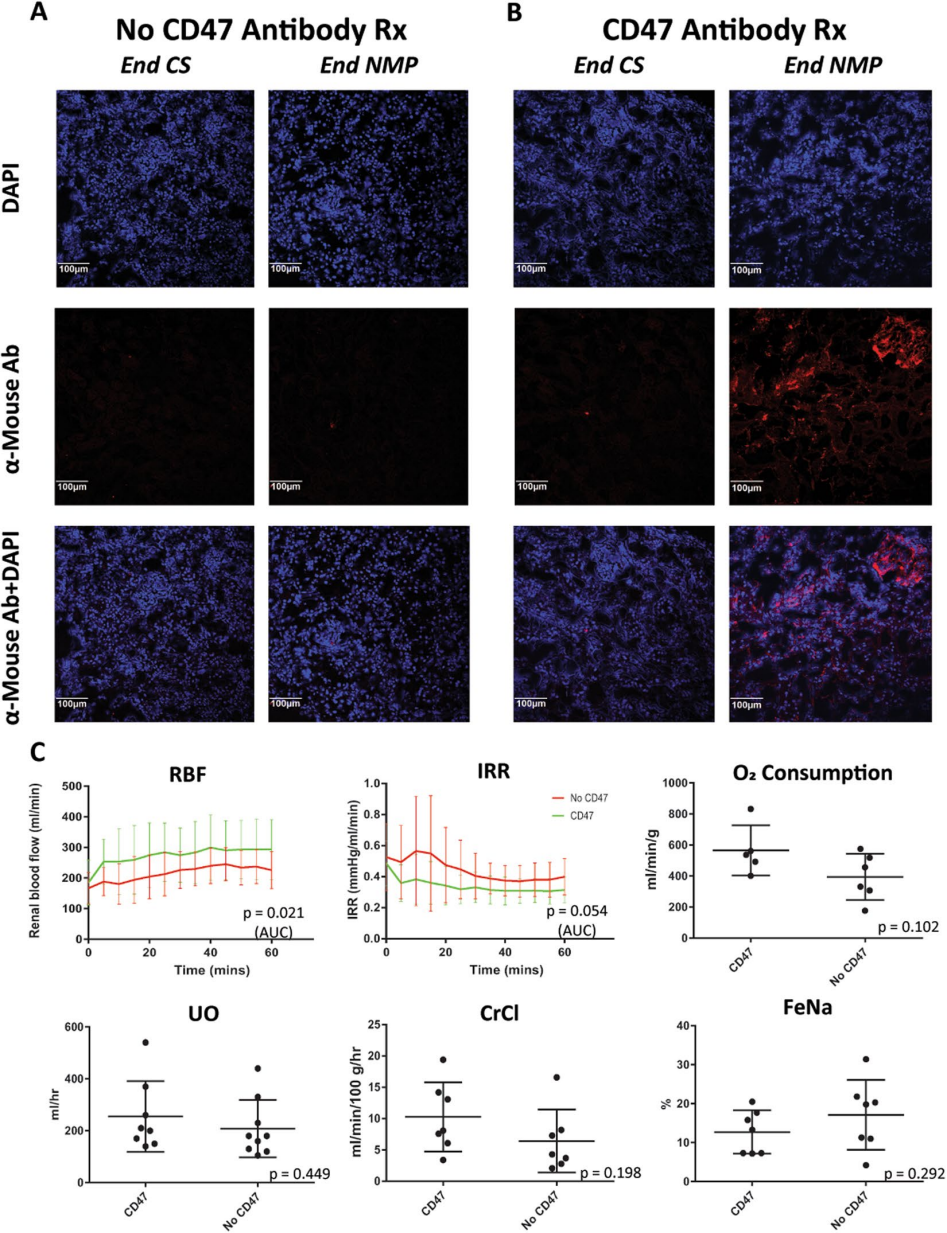
(**A**,**B**) αCD47Ab localisation in porcine NMP kidneys by immunofluorescence (20×). αCD47Ab was given to treatment group kidneys by addition of the drug into the (i) UW cold flush, and (ii) NMP circuit. (**A**) No antibody binding evident in control kidneys (i.e. kidneys without addition of αCD47Ab during NMP). (**B**) Faint/minimal antibody binding at the end of CS (i.e. prior to the commencement of NMP); strong binding is evident in biopsies at the end of NMP, especially in the glomerulus. (**C**) Flow, IRR, glomerular, and tubular parameters after 1 hr of NMP in porcine kidneys treated with αCD47Ab in comparison to NMP without CD47 treatment. Data presented as mean ± SD; n = 8–9/group. AUC – area under the curve; CrCl – creatinine clearance; CS – cold storage; FeNa – fractional excretion of sodium; NMP – normothermic machine perfusion; UO – urine output; UW – University of Wisconsin solution.

There was no αCD47Ab binding evident in untreated kidneys (Fig. 6A). In the treated kidneys, addition of αCD47Ab to the UW cold flush did not result in binding of the antibody to the kidney (Fig. 6B, ‘End CS’). In contrast, direct antibody infusion into the arterial line at the commencement of NMP resulted in widespread αCD47Ab binding along the glomerulus and renal tubular epithelium, which was detectable at the end of NMP (Fig. 6B, ‘End NMP’).

#### αCD47Ab treatment during NMP improves renal perfusion parameters

In comparison to untreated kidneys, kidneys receiving αCD47Ab during NMP had greater RBF and lower IRR (Fig. 6C). However, renal oxygen consumption, UO, CrCl, and FeNa were not statistically different (Fig. 6C).

#### Pro-inflammatory cytokine mRNA expression is increased after NMP

**Figure 7:**
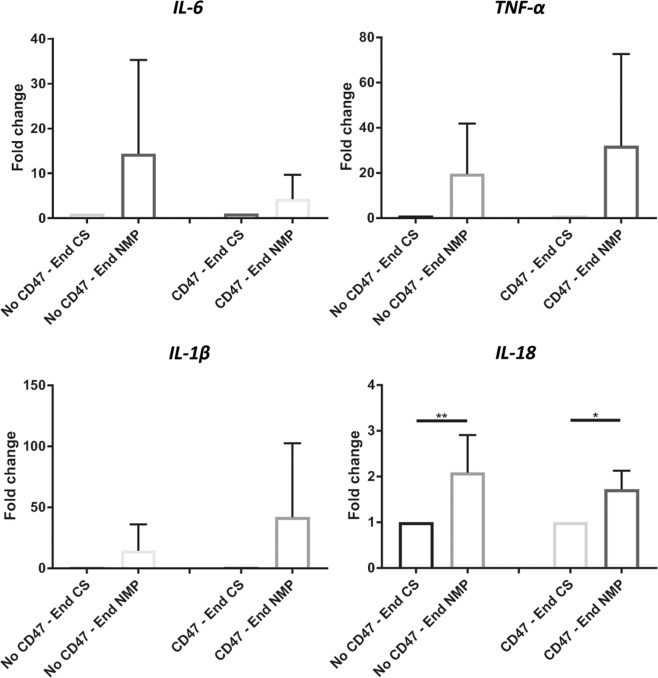
Pro-inflammatory cytokine (IL-6, TNF-α, IL-1β, and IL-18) mRNA expression in porcine tissue from sections taken at the end of CS and then following the end of NMP in kidneys with and without αCD47Ab treatment. Fold change normalized to HPRT1. Data shown as mean ± SD; n = 7/group. *p < 0.05, **p < 0.01.

In both treated and untreated kidneys, renal expression of IL-6, TNF-α, IL-1β, and IL-18 increased after NMP in comparison to samples taken at end CS (i.e. immediately preceding NMP), but this only reached significance for IL-18 (Fig. 7). In congruence with the mouse RT-PCR data, expression levels of TNF-α and IL-1β was elevated in the αCD47Ab treatment group, although the significance of this was not clear.

#### Renal tubular debris is reduced in CD47-treated kidneys after NMP but other histologic parameters remain similar to controls

**Figure 8:**
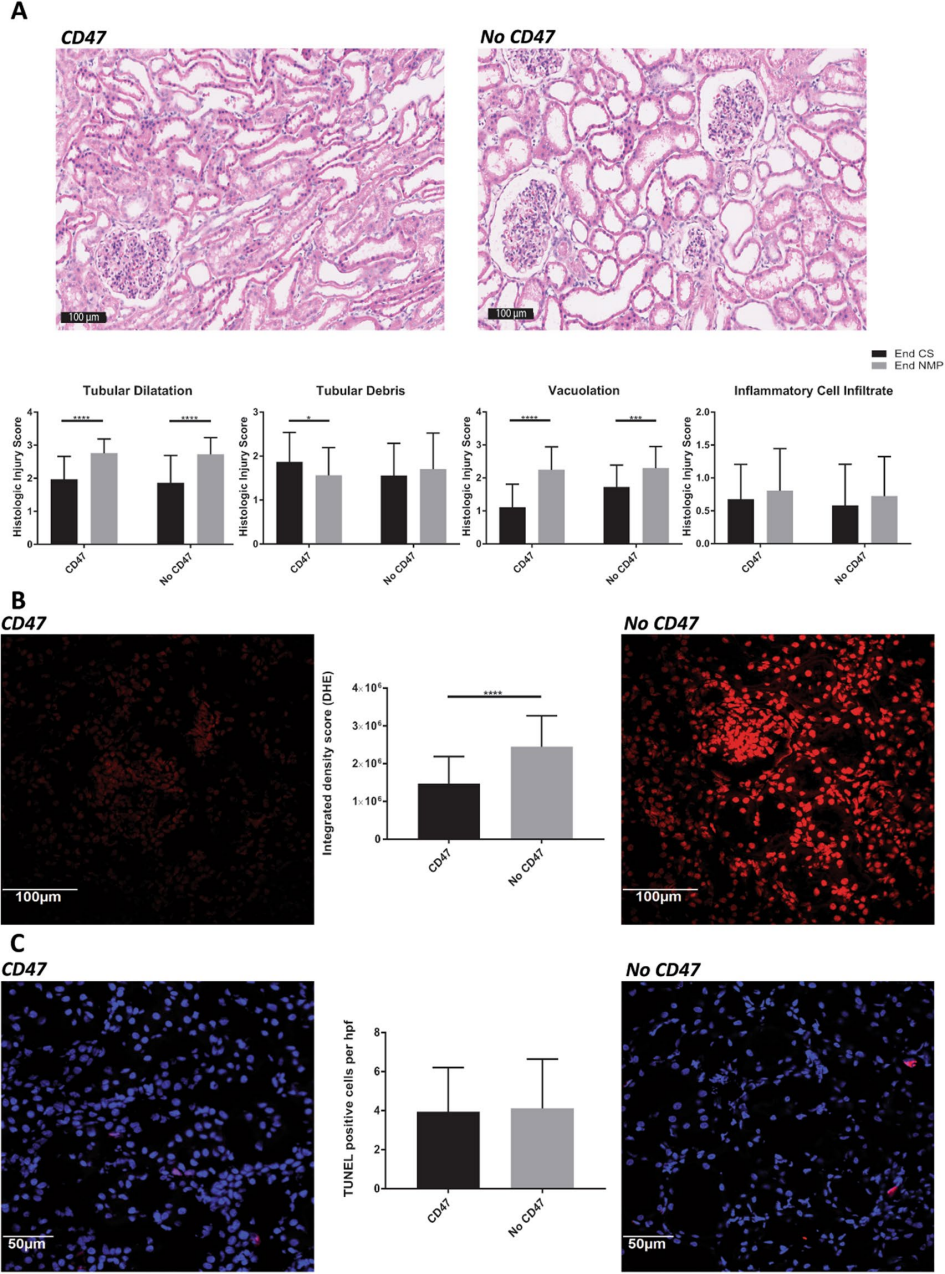
Representative porcine renal tissue photomicrographs and quantitative scoring for (**A**) tubular injury (H&E staining) (20×), (**B**) oxidative stress (DHE staining) (20×), and (**C**) renal cellular death (TUNEL staining; 40×) at the end of NMP. Data shown as mean ± SD; n = 7/group. *p < 0.05, ***p < 0.001, ****p < 0.0001.

NMP re-institutes oxygenated blood flow to the kidney after a period of cold ischemia, and as such would be expected to precipitate IRI, albeit at a reduced magnitude due to the leukocyte depletion of the blood. Histologic comparison of the renal tubular condition before and after NMP showed a significant increase in tubular dilatation and vacuolation in both treated and untreated kidneys (Fig. 8A). There was also no significant change in inflammatory cell infiltrates. However, there was a significant decrease in tubular debris in the αCD47Ab-treated kidneys after NMP.

#### Renal oxidative stress induced by NMP is reduced by αCD47Ab treatment however renal tubular epithelial cell death remained similar

Oxidative stress quantified by DHE staining was significantly reduced in αCD47Ab-treated kidneys in comparison to controls (Fig. 8B). Renal cellular death however was not significantly different between treated and untreated kidneys (Fig. 8C).

## Discussion

This study provides the first direct *in vivo* comparison of three potent anti-IRI agents, αCD47Ab, sCR1, and rTM, in a murine model of severe renal IRI. We show that αCD47Ab alone provides the greatest level of protection that is not substantially increased by combination with sCR1. We successfully demonstrated delivery of αCD47Ab to porcine kidneys using NMP, with evidence of renal tubular and glomerular binding, and subsequent downstream beneficial effects on kidney perfusion and oxidative stress.

CD47 provides a plausible target that can be blocked to significantly ameliorate IRI via the modulation of multiple IRI-related pathways. CD47 signaling is important for promoting IRI; injury primarily results from the renal parenchymal cell membrane-associated CD47 binding its ligand TSP1^21^. Downstream effects include inhibition of nitric oxide and its effects on vascular smooth muscle, exacerbation of oxidative stress, inflammatory cell recruitment, and an impairment of parenchymal cellular repair^21,30,46^. As such, receptor blockade should ameliorate IRI by impacting multiple inter-related injurious processes. In our mouse model, we confirmed a significant reduction to renal injury, with better preservation of renal function in the αCD47Ab treated mice, which was superior to that provided by sCR1 or rTM alone. Although the reduction in BUN and creatinine levels in the αCD47Ab group were relatively small, this was in the context of severe renal IRI and comparatively better protection than other drugs utilized.

Neutrophil influx was also correspondingly reduced in the αCD47Ab group, in addition to a robust decrease in renal cellular death. Although levels of superoxide significantly declined in the αCD47Ab group, no reduction was seen in hydrogen peroxide levels. Indeed, substantial fluxes of superoxide may significantly impact the stoichiometry of hydrogen peroxide detection by amplex red, possibly explaining the different quantification trends of superoxide and hydrogen peroxide in the study groups^47^. Interestingly, with the exception of IL-6, there was no reduction in the mRNA expression of TNF-α, IL-1β, CCL2, or CXCL2 in the αCD47Ab-treated mice. A lack of impact on TNF-α mRNA expression in response to CD47 blockade has also been noted elsewhere, indicating this pathway is not directly involved in CD47-mediated cellular injury during IRI^21,48^.

The primary focus of this paper was the acute phase of injury secondary to IRI, which manifests as delayed graft function (DGF) in the kidney transplant recipient. However, it is also important to demonstrate the potential efficacy of CD47 blockade in the prevention of progression of AKI to chronic kidney disease (CKD) and prove that αCD47Ab does not merely delay the onset of AKI. Our group therefore undertook additional work that showed αCD47Ab administered to C57BL/6 mice in an ischemic model of CKD limited the development of fibrosis, supporting the hypothesis that targeting this pathway can also limit AKI-to-CKD transition^49^.

In order to investigate the potential synergistic amelioration of IRI by combining different drugs, the two most efficacious drugs, αCD47Ab and sCR1, were given in combination. Although serum creatinine levels were significantly lower in these mice compared to sCR1 alone, there was no significant difference between the αCD47Ab + sCR1 group and the group of mice treated with αCD47Ab alone. Furthermore, histologic injury, inflammatory cell infiltration, complement deposition, ROS production, and cellular death were not incrementally improved in the combined treatment group compared to αCD47Ab-treated mice. This acts to highlight the relatively broad impact of CD47-blockade on the IRI cascade, therefore serving as a highly effective single agent.

CD47Ab was chosen as the optimal agent to be administered using NMP in porcine kidneys. Antibody was retained in the renal parenchyma at the end of NMP, ensuring the CD47 receptor remains blocked prior to potential transplantation. The drug was dosed according to kidney weight and not the weight of the donor animal, as NMP affords the opportunity of direct intra-renal delivery. There was no immunofluorescence evidence that this cold perfusion component of the αCD47Ab dose caused effective binding to its receptor. In contrast, Xu *et al*. showed renal binding after pre-implantation delivery of αCD47Ab to porcine kidneys via a direct renal artery cold flush^48^. However, these authors used a dose that was approximately 50 times greater than that used in this study. We opted for a lower dose owing to its efficacy in the murine model in addition to significant costs associated with larger doses of this antibody^48^.

Addition of αCD47Ab to the NMP perfusion circuit did not alter renal injury on the machine in comparison to control kidneys. NMP involves reperfusion of the kidney with an oxygenated red blood cell based-based solution, and as such can be considered as an early induction of IRI after a period of CS. The primary difference between NMP and reperfusion after transplantation is that the latter occurs in a uremic recipient with allogeneic whole blood containing the recipient’s leukocytes, pre-formed antibodies, and complement components. As such, the insult sustained during NMP is unique in its nature. Overall, a pro-inflammatory state is induced^50,51^. Therefore, and unsurprisingly, renal mRNA expression of pro-inflammatory cytokines increased after NMP in this study, albeit to a lesser extent for IL-6 and IL-18 in the αCD47Ab-treated group. Furthermore, there was a mild increase in renal tubular injury parameters as evident by light microscopy post-NMP; these parameters were similar in both groups with the exception of tubular debris, which was significantly reduced in the CD47-blocked group. Importantly, the oxidative stress induced by NMP was significantly less in the αCD47Ab treatment group.

CD47 blockade during NMP enhanced some functional parameters over the course of perfusion. RBF and IRR were significantly better in the treatment group, which may be related to the effects of CD47 binding on nitric oxide and vascular responsiveness^46^. Improvements to additional parameters during NMP might require a higher dose of αCD47Ab or the induction of more severe injury through prolongation of ischemic times. Furthermore, any ultimate improvement in renal IRI by CD47 blockade needs to be proven after full-scale reperfusion with leukocyte-replete allogeneic blood (i.e. transplantation).

Pharmacomanipulation of the kidney during NMP may also improve the efficacy of the short periods of pre-implantation NMP currently in clinical use^12^. There is some experimental evidence to indicate the longer periods (8 or more hours) of renal NMP are superior to 1 hour of pre-implantation NMP; this is in the setting where no additional anti-IRI drugs are added^52,53^. Longer periods of NMP are however more labor-intensive, expensive, and likely less readily taken up by transplant centers. Pharmacologic amelioration of IRI during NMP may provide a compromise, allowing shorter pre-implantation NMP.

In conclusion, this paper has shown the feasibility and efficacy of using NMP as a targeted drug delivery system to the kidney as a means to ameliorate IRI. Three proven anti-IRI drugs were compared in a murine kidney model of severe IRI, and αCD47Ab was shown to be most protective. The porcine-specific version of this antibody was tested in a DCD model using NMP, achieving renal binding, and improving some renal perfusion and injury parameters. NMP has a remarkable potential to not only directly treat and resuscitate donor kidneys prior to implantation, but also to fast-track drug discovery/application from small animal and/or cell culture models into the clinical setting. Its impacts may be significantly amplified through the targeted delivery of anti-IRI drugs to the kidney, which will likely translate into vast future clinical applications.

## Supplementary information

Supplementary information

## Data Availability

The datasets generated during and/or analysed during the current study are available from the corresponding author on reasonable request.
