# Supplementary material for: Pharmacologic targeting of renal ischemia-reperfusion injury using a normothermic machine perfusion platform
Source: Sci Rep. 2020 Apr 24;10:6930. doi: 10.1038/s41598-020-63687-0 (PMC7181764; doi:10.1038/s41598-020-63687-0)
Supplement: Supplementary file 1 — Supplementary information [file 41598_2020_63687_MOESM1_ESM.docx]

**Pharmacologic targeting of renal ischemia-reperfusion injury using a normothermic machine perfusion platform**

**Authors**: Ahmer M. Hameed (MBBS, MS) ^1,2,3^, David B. Lu (MD, PhD)^2^, Heather Burns (BSc)^2^, Nicole Byrne (BVSc)^2^, Yi Vee Chew (PhD)^2^, Sohel Julovi (PhD) ^2^, Kedar Ghimire (PhD)^2^, Negar Talaei Zanjani (PhD)^2^, Chow H. P’ng (MBBS, FRCPA)^4^, Daniel Meijles (PhD)^5^, Suat Dervish (PhD)^2^, Ross Matthews (BVSc)^6^, Ray Miraziz (MSc)^7^, Greg O’Grady (FRACS, PhD)^8^, Lawrence Yuen (FRACS)^1,3^, Henry C. Pleass (FRACS, MD)^1,3^, Natasha M. Rogers* (FRACP, PhD)^2,3,9^, and Wayne J. Hawthorne* (MD, PhD)^1,2,3^

**Affiliations**:

1. Department of Surgery, Westmead Hospital, Sydney, Australia
2. Westmead Institute for Medical Research, Sydney, Australia
3. Sydney Medical School, University of Sydney, Australia
4. Institute for Clinical Pathology and Medical Research, Westmead Hospital, Sydney, Australia
5. St George’s, University of London, UK
6. Department of Animal Care, Westmead Hospital, Sydney, Australia
7. Department of Anesthesia, Westmead Hospital, Sydney, Australia
8. Department of Surgery, The University of Auckland, Auckland, New Zealand
9. Department of Transplant/Renal Medicine, Westmead Hospital, Sydney, Australia

* Co-senior authors


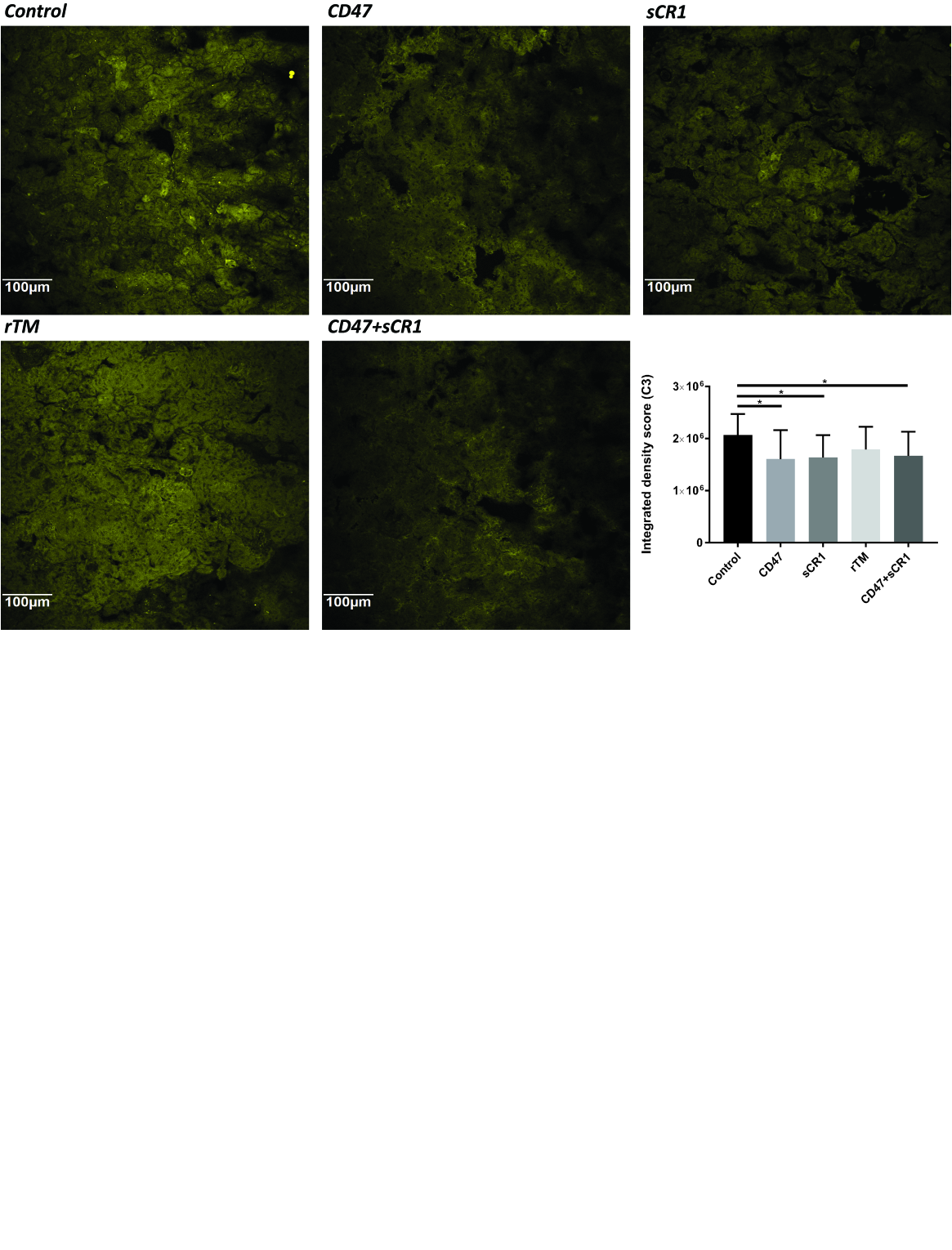
**Supplemental Digital Content** 1**.** Complement C3 staining in all murine study groups 24 hrs of induction of ischemia-reperfusion injury, as visualized by immunofluorescence (20 x). Data shown as mean ± SD; n = 5-6/group. *p<0.05.
